# Cancer-testis antigen MAGE-C2 binds Rbx1 and inhibits ubiquitin ligase-mediated turnover of cyclin E

**DOI:** 10.18632/oncotarget.5973

**Published:** 2015-10-19

**Authors:** Jiaqing Hao, Xiao Song, Jingjing Wang, Chengli Guo, Yan Li, Bing Li, Yu Zhang, Yanhui Yin

**Affiliations:** ^1^ Department of Immunology, School of Basic Medical Sciences, Peking University Health Science Center, Beijing, China; ^2^ Department of Microbiology and Immunology, University of Louisville, Louisville, Kentucky, USA

**Keywords:** cancer-testis antigen, MAGE-C2, SCF complex, cyclin E, cell cycle

## Abstract

Cancer-testis antigen MAGE-C2 is normally expressed in testis but aberrantly expressed in various kinds of tumors. Its functions in tumor cells are mostly unknown. Here, we show that MAGE-C2 binds directly to the RING domain protein Rbx1, and participates in Skp1-Cullin1-F box protein (SCF) complex. Furthermore, MAGE-C2 can inhibit the E3 ubiquitin ligase activity of SCF complex. Ablation of endogenous MAGE-C2 decreases the level of cyclin E and accelerates cyclin E turnover by inhibiting ubiquitin-mediated proteasome degradation. Overexpression of MAGE-C2 increases the level of cyclin E and promotes G1-S transition and cell proliferation, and the results are further confirmed by knockdown of MAGE-C2. Overall, the study indicates that MAGE-C2 is involved in SCF complex and increases the stability of cyclin E in tumor cells.

## INTRODUCTION

Cancer-testis antigen MAGE-C2 was identified by Boon's and our groups [1–2], belonging to type I MAGE (melanoma antigen) family. Type I MAGE cancer-testis antigens represent ideal targets for cancer vaccines due to their immunogenicity in cancer [3–6], and some clinical trials with MAGE peptides have achieved encouraging results in cancer patients [7–9]. However, for quite a long time, it remains unclear whether MAGE expression in cancer cells is a non-functioning by-product of cellular transformation or actually contributes to the development of malignancies [10]. Recent studies have explored the roles of MAGE proteins in cancer cells, and they were observed to promote cancer cell survival, tumor formation, and metastasis [11–13]. More recently, Doyle JM et al showed that multiple MAGE proteins can form complexes with RING domain proteins in cells, such as MAGE-A2/C2-TRIM28, MAGE-B18-LNX1 complexes, etc [13]. Rbx1 (RING Box Protein-1) is a RING component of the largest E3 ligases SCF complex [14]. SCF complex consists of Rbx1, Cullin1, Skp1 and F-box protein family. The Cullin1/Rbx1 components form the core of E3 ligase that associates with E2 ubiquitin conjugating enzymes, Skp1 serves as an adapter that links Cullin1 to the variable F-box protein which functions in substrate targeting. It has been reported that deregulation of SCF-dependent proteolysis can cause a variety of diseases including cancer [15–16].

As one of the most immunogenic cancer-testis antigen, MAGE-C2 is expressed at a high frequency in various kinds of tumors [1, 17–19]. Previous work was mainly focused on the development of cancer vaccines toward MAGE-C2 due to its specific expression in spermatogonia germ cells and cancers [18, 20–22], however, the biological function of MAGE-C2 in both the germ line and tumors has remained poorly understood. In this study, we demonstrate that cancer-testis antigen MAGE-C2 binds directly to the RING domain containing protein Rbx1, inhibits ubiquitin-dependent degradation of cyclin E, and promotes cell cycle progression at G1-S transition.

## RESULTS

### MAGE-C2 binds to Rbx1 and Cullin1

**Figure 1:**
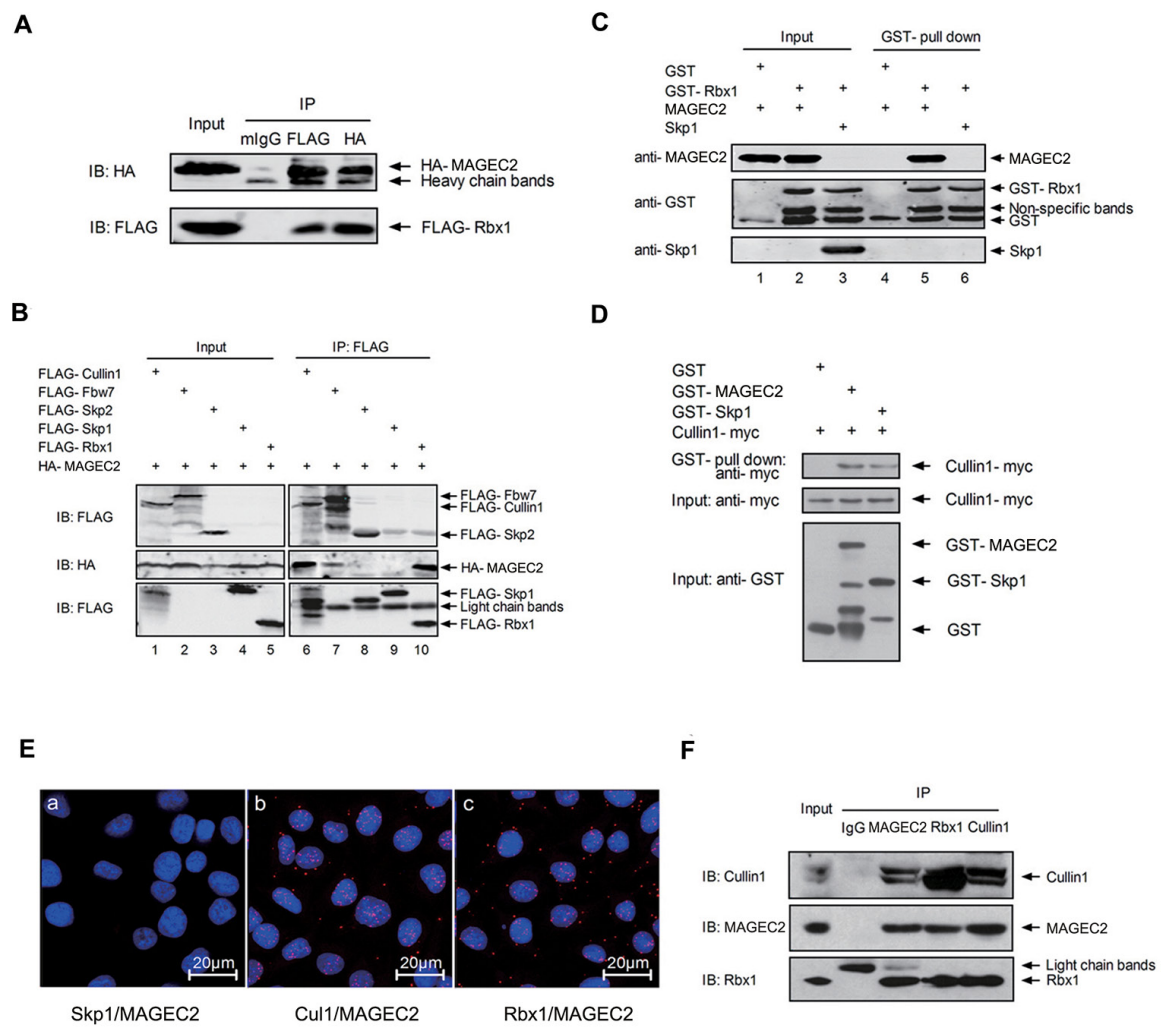
MAGE-C2 binds with Rbx1 and Cullin1 **A.** MAGE-C2 binds with Rbx1. Lysates from HEK293T cells co-transfected with HA-MAGE-C2 and FLAG-Rbx1 were immunoprecipitaed with anti-FLAG, anti-HA antibodies or control mouse IgG followed by immunoblotting with indicated antibodies. **B.** MAGE-C2 binds with Cullin1 and Fbw7. HEK293T cells were transfected as indicated at the top, immunoprecipitated with anti-FLAG antibody, and bound proteins were detected by immunoblotting with anti-FLAG or anti-HA antibodies. **C.** MAGE-C2 binds Rbx1 directly. GST pull-down experiment was performed with GST-fused Rbx1 or GST (a negative control) and purified MAGE-C2 or Skp1 (as a negative control). Proteins were detected with Western blotting. **D.** MAGE-C2 binds Cullin1 directly. GST pull-down experiment was performed with the *in vitro* translated Cullin1 and GST-MAGE-C2, GST-Skp1 (a positive control) or GST. Proteins were detected with Western blotting. **E.** Associations of MAGE-C2 with Cullin 1 and Rbx1 were visualized in A375 cells with an *in situ* proximity ligation assay. The interaction was visualized as red fluorescent spots. The cells were counterstained with Hoechst (blue) to visualize the nuclei. **F.** Endogenous MAGE-C2, Rbx1 and Cullin1 bind with each other. Immunoblot analysis of SK-mel-37 cell lysates and the immunoprecipitates with indicated antibodies. Rabbit IgG was used as a negative control for the immunoprecipitation.

Recent evidence indicates that a general property conserved in the MAGE family is their binding to E3 RING proteins [13]. This leads us to speculate whether MAGE-C2 can bind to Rbx1, a RING component of SCF complex. To study this possibility, expression plasmids for HA-MAGE-C2 and FLAG-Rbx1 were cotransfected into HEK293 T cells, and co-immunoprecipitation assay was performed. As shown in Figure 1A, MAGE-C2 was detected in immunoprecipitates with the anti-FLAG antibody, and alternatively, FLAG-Rbx1 was detected in immunoprecipitates with the anti-HA antibody. These results showed that MAGE-C2 could bind with Rbx1. To further test whether MAGE-C2 could also bind to the other core components of SCF complex, we coexpressed HA-MAGE-C2 and FLAG-tagged Cullin1, or FLAG-tagged F-box proteins (Fbw7 or Skp2), or Skp1 (Rbx1 as a positive control) in HEK293 T cells, respectively, and performed co-immunoprecipitation studies (Figure 1B). HA-MAGE-C2 was specially detected in FLAG-Cullin1, FLAG-Fbw7 and FLAG-Rbx1 immunoprecipitates (lanes 6, 7 and 10), but not in FLAG-Skp1 or FLAG-Skp2 immunoprecipitates (lanes 8 and 9), indicating that MAGE-C2 binds to Cullin 1 and Fbw7, but not Skp1 or Skp2.

To determine whether MAGE-C2 directly bind the components of SCF complex, we purified recombinant GST-Rbx1, GST-MAGE-C2, and GST-Skp1 from bacteria and translated the Cullin1-myc protein *in vitro*. GST pull-down experiments indicated that MAGE-C2 can bind to Rbx1 (Figure 1C, lane 5) and Cullin1 (Figure 1D), but not Skp1 (Supplementary Figure S1).

To examine whether endogenous MAGE-C2 binds to endogenous Cullin1 and Rbx1, we firstly investigated the subcellular localization of MAGE-C2 with immunostaining A375 cell. MAGE-C2 was localized in the nucleus (Supplementary Figure S2), consistent with the nuclear localization of Cullin1 and Rbx1 [23]. Then we applied *in situ* proximity ligation assay (PLA) in A375 cells. MAGE-C2 binds to Rbx1 and Cullin1, but not Skp1 as evidenced by the presence of multiple associated dots appearing mostly in the nucleus (Figure 1E). Moreover, endogenous bindings of MAGE-C2 with Rbx1 and Cullin1 were further confirmed by co-immunoprecipitation analysis in SK-Mel-37 cells (Figure 1F). These results suggest that MAGE-C2, Rbx1 and Cullin1 bind to each other within cells.

### MAGE-C2 is involved in SCF complex

**Figure 2:**
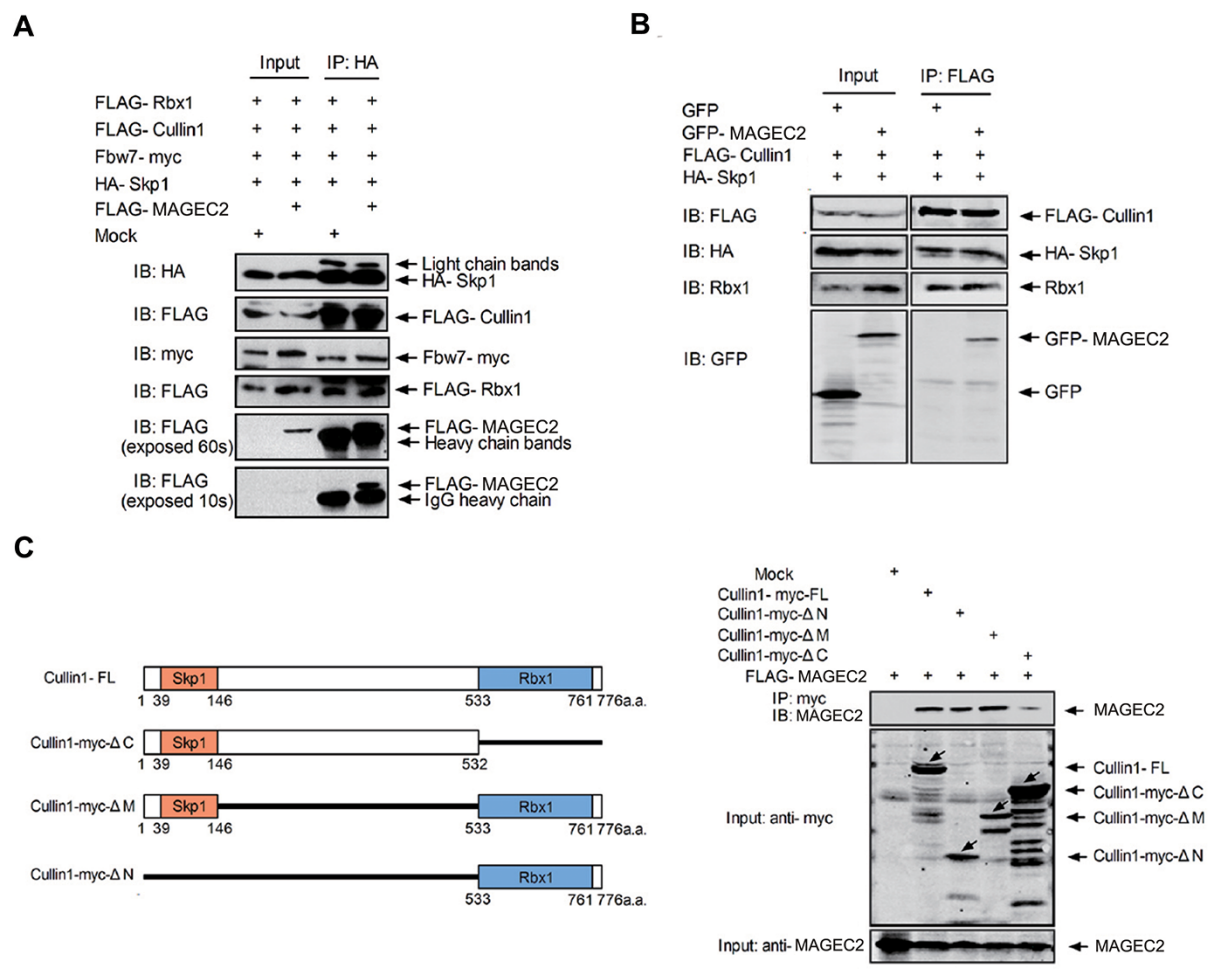
MAGE-C2 participates in SCF complex and does not interfere with binding of Skp1 and Rbx1 to Cullin1 **A.** MAGE-C2 participates in the Skp1-Cullin1-F box protein complex. Lysates from HEK293T cells transfected with plasmids as indicated were immunoprecipitated with anti-HA antibody and immunoblotted with anti-HA, anti-FLAG or anti-myc antibodies. **B.** MAGE-C2 does not interfere with binding of Skp1 and Rbx1 to Cullin1. Lysates from HEK293T cells transfected with plasmids as indicated were immunoprecipitated with anti-FLAG antibody followed by immunoblotting with indicated antibodies. **C.** Binding region of Cullin1 to MAGE-C2. FL or deletion mutants of myc-tagged Cullin1 were cotransfected with FLAG-MAGE-C2 into HEK293T cells. Lysates were subjected to immunoprecipitation with anti-myc antibody following immunoblotting with anti-myc or anti-MAGE-C2 antibodies.

Since MAGE-C2 directly binds with Rbx1 and Cullin1, but not Skp1, we asked whether MAGE-C2 exists in the Rbx1-Cullin1-Skp1-F-box protein complex. To test this, HEK293 T cells were transfected with expression constructs of FLAG-tagged Rbx1, Cullin1, and MAGE-C2, Fbw7-myc, and HA-Skp1. As shown in Figure 2A, FLAG-tagged MAGE-C2, Rbx1 and Cullin1, myc-tagged Fbw7 were all detected in HA-Skp1 immunoprecipitates, suggesting that MAGE-C2 is involved in the Cullin-Skp1-Fbw7 complex.

As Cullin1 is a scaffold component with its amino terminus binding to Skp1 and the carboxyl terminus with Rbx1, we examined whether the binding of MAGE-C2 with Cullin1 interferes the binding of Skp1 and Rbx1 to Cullin1. HEK-293T cells were transfected with constructs of FLAG-Cullin1, HA-Skp1, and GFP-MAGE-C2 or GFP, and co-immunoprecipitation analysis indicated that HA-Skp1, GFP-MAGE-C2, and endogeneous Rbx1 were all existed in FLAG-Cullin1 immunoprecipitates (Figure 2B). These data showed that MAGE-C2 does not disrupt the SCF complex formation of Cullin1.

We further assessed the structural requirements for MAGE-C2-Cullin1 complex formation with various deletion mutants of Cullin1. We tested the bindings of MAGE-C2 with Cullin1-myc-ΔN (lacking the N-terminal 532 amino acid residues), Cullin1-myc-ΔC (lacking the C-terminal 243 amino acid residues), and Cullin1-myc-ΔM (lacking residues 148 to 532). As shown in Figure 2C, C-terminal region (residues 533 to 776) of Cullin1 is required for binding with MAGE-C2. To map Cullin1/Rbx1 binding domain on MAGE-C2, a panel of MAGE-C2 deletion mutants were cotransfected with Cullin1 or Rbx1 into HEK293T cells. Neither deletion of MHD domain (MAGE-C2 Δ148–314), deletion of N-terminus (MAGE-C2 Δ31–147), or deletion of C-terminus (MAGE-C2 Δ245–373) abrogated the binding of Cullin1/Rbx1 to MAGE-C2 (Supplementary Figure S3), indicating that there are multiple Cullin1/Rbx1 binding sites on MAGE-C2.

### MAGE-C2 inhibits E3 ubiquitin ligase activity

**Figure 3:**
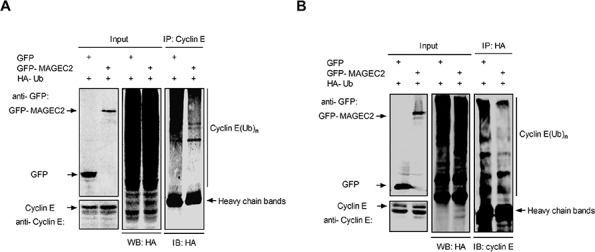
MAGE-C2 inhibits E3 ubiquitin ligase activity **A.** MAGE-C2 inhibits ubiquitylation of cyclin E *in vivo*. HEK293T cells were cotransfected with HA-Ub together with GFP-MAGE-C2 or GFP. At 36 h after transfection, the cells were treated with 20 μM MG132 for 8 h, and then lysed and immunoprecipitated with anti-cyclin E followed by immunoblotting with anti-HA. **B.** The cell lysates prepared as (A) were immunoprecipitated with anti-HA and immunoblotted with anti-cyclin E.

To determine the effect of MAGE-C2 on the ubiquitin ligase activity of SCF complex, we examined the ubiquitylation of cyclin E in the presence or absence of MAGE-C2. HA-ubiquitin and GFP-MAGE-C2 or GFP expression plasmids were cotransfected into HEK-293T cells, and MG-132 was used to enrich the ubiquitinated species in cells. Cell extracts were subjected to immunoprecipitate with anti-cyclin E or anti-HA antibodies, and copurified proteins were probed by immunoblotting with indicated antibodies. We observed that transfection with GFP-MAGE-C2 significantly reduced the amount of ubiquitylation of cyclin E compared to transfection with GFP (Figure 3A and 3B).

### MAGE-C2 increases cyclin E stability in cells

**Figure 4:**
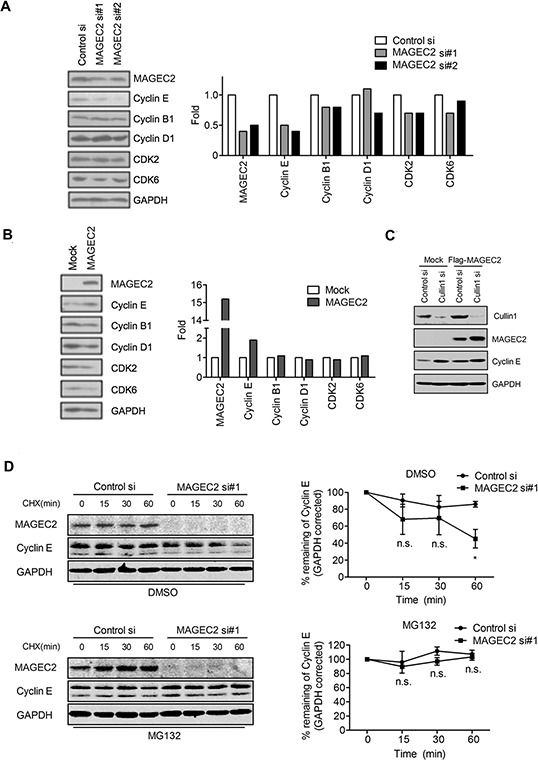
MAGE-C2 regulates cyclin E stability in cells **A.** MAGE-C2 knockdown decreases cyclin E protein levels. MAGE-C2 siRNA or control siRNA was transfected into A375 cells. Lysates were prepared 48 h after transfection and immunoblotted with anti-MAGE-C2 antibody. Cyclin E, cyclin B1, cyclin D1, CDK2, CDK6 and GAPDH protein levels were analyzed by immunoblotting with indicated antibodies. Expression levels of GAPDH are indicated as an internal control. The quantitative data is shown in right graphs. **B.** Overexpression of MAGE-C2 increases cyclin E protein levels. HEK293T cells were transfected with MAGE-C2-expressing or control (mock) vectors and lysates were prepared 48 h after transfection. MAGE-C2, cyclin E, cyclin B1, cyclin D1, CDK2, CDK6 and GAPDH protein levels were analyzed by immunoblotting with indicated antibodies. **C.** Cullin1 is required for MAGE-C2 induced enhancement of cyclin E. FLAG-MAGE-C2 and Cullin1 siRNA were cotransfected into HEK293T cells, and cells were harvested at 48 h after transfection. Immunoblotting were performed with indicated antibodies. **D.** Downregulation of MAGE-C2 decreased cyclin E protein half-life. MAGE-C2 siRNA or control siRNA was transfected into A375 cells. Twenty-four hours after transfection, cells were treated with vehicle (DMSO) or MG132 (20 μM) for 8 h before addition of CHX (50 μg/ml). Cells were harvested for Western blotting at the indicated time. The results were plotted after quantitation (right).

Next, we investigated the stability of cyclin E in cells. MAGE-C2 siRNAs induced a decrease in intracellular cyclin E in MAGE-C2-positive A375 cells (Figure 4A) compared with control siRNA. In addition, overexpression of MAGE-C2 in HEK-293 T cells increased endogenous cyclin E levels (Figure 4B). To investigate if Cullin1 was involved in this process, Cullin1 siRNA and Flag-MAGE-C2 were transfected into HEK293T cells. As expected, upregulation of MAGE-C2 expression following Cullin1 knockdown did not increase cyclin E level (Figure 4C, lane 4 compared with lane 2), indicating that Cullin1 contributes to the enhancement of cyclin E induced by MAGE-C2 expression. There were no significant changes for cyclinB1, cyclinD1, CDK2 and CDK6 by overexpression or knockdown of MAGE-C2 (Figure 4A and 4B). To exclude the possibility that MAGE-C2 increases mRNA levels, we performed qRT-PCR on RNA prepared from MAGE-C2-depleted or MAGE-C2-overexpression cells, and the result showed no significant changes (Supplementary Figure S4).

To analyze the effect of MAGE-C2-depletion on cyclin E turnover, we did a cycloheximide (CHX) assay. As shown in Figure 4D, the results indicated that ablation of MAGE-C2 in A375 cells shortened the half-life of cyclin E protein, and this process was blocked by MG132 treatment.

These findings demonstrate that MAGE-C2 increases cyclin E stability in proteasome-dependent manner.

### MAGE-C2 promotes the progression through the G1 to S phase

**Figure 5:**
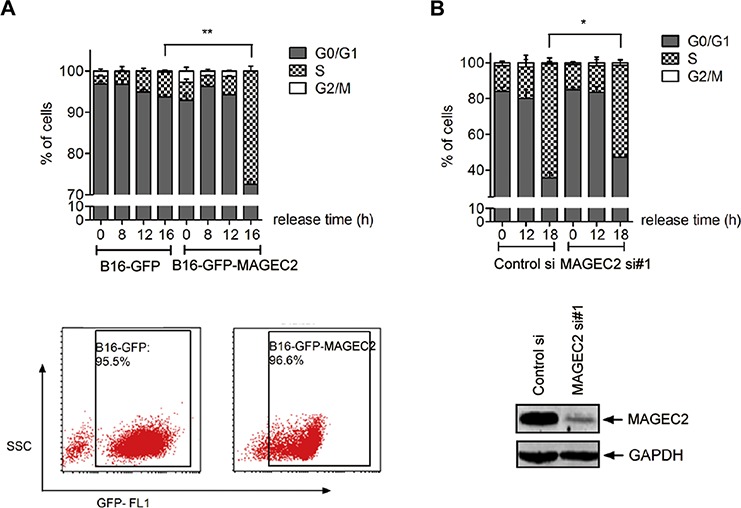
MAGE-C2 promotes the progression through the G1 to S phase **A.** Overexpression of MAGE-C2 promotes the cell cycle progression from G1-S phase. Stable MAGE-C2-overexpressing B16 cells were synchronized at G1 phase by lovastatin for 34 h and then cultured in a medium containing 2 mM mevalonic acid. At the indicated periods thereafter, cells were harvested and stained with PI for cell cycle analysis. MAGE-C2 expression was confirmed by FACS (bottom). **B.** Downregulation of MAGE-C2 attenuates cell cycle progression from G-S phase. A375 cells transfected with MAGE-C2 siRNA were synchronized and analyzed as in (A). Downregulation of MAGE-C2 was confirmed by Western blotting (bottom).

Since cyclin E has been considered an essential regulator of cell cycle transition from G1 to S, the finding that MAGE-C2 inhibits cyclin E degradation prompted us to investigate the role of MAGE-C2 in G1 to S phase transition in tumor cells. We used lovastatin for cell synchronization to investigate the transition from the G1 to S phases. Stable tranfectants of MAGE-C2 (B16-GFP-MAGE-C2) and control cells expressing GFP were cultured with lovastatin for 34 hours, then replaced with mevalonic acid and cells were collected at 8 hr, 12 hr and 16 hr, respectively. FACS analysis showed MAGE-C2 expressing transfectants progressed more quickly through G1 phase compared to the control cells. At 16 hr, 27.49 ± 2.00% of B16-MAGE-C2 cells entered S phase, while only 5.94 ± 0.78% of B16-GFP cells were in S phase (Figure 5A).

To confirm the role of MAGE-C2 on promoting G1-S progression, we used MAGE-C2 specific siRNAs. A375 cells were synchronized in G1 phase with lovastatin following siRNA transfection, and were harvested at 12 hr and 18 hr after adding mevalonic acid respectively. As shown in Figure 5B, A375 cells treated with MAGE-C2 siRNA hindered the cells from going into the S phase, as indicated by the reduced percentage of cells entering S phase compared with control siRNA (51.32 ± 5.68% vs. 63.51 ± 5.90%).

### MAGE-C2 promotes cell proliferation

**Figure 6:**
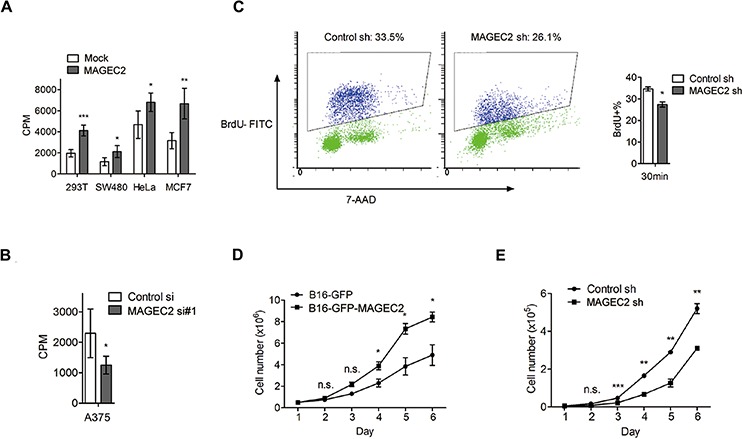
MAGE-C2 promotes cell proliferation **A.** Overexpression of MAGE-C2 increased DNA synthesis. HEK293T, SW480, HeLa and MCF7 cells were transfected with MAGE-C2-expressing or control (mock) vectors. Seventy-two hours later, ^3^H-thymidine incorporation was measured as described under Materials and Methods. The mean and standard deviation of triplicate measurements are shown. **B.** Knockdown of MAGE-C2 suppressed DNA synthesis in A375 cells, as determined by ^3^H-thymidine incorporation assay. **C.** MAGE-C2 stable knockdown suppressed DNA synthesis in A375 cells, as determined by BrdU incorporation assay. **D.** Stable overexpression of MAGE-C2 significantly promoted B16 cell growth in the course of 6 days. The cell growth was monitored by counting cells at indicated times. **E.** Stable knockdown of MAGE-C2 inhibited A375 cell growth, as determined by counting cell numbers. The mean and standard deviation of triplicate measurements are shown.

Next, we assessed the effect of MAGE-C2 on cell proliferation with [^3^H] thymidine incorporation assay. As shown in Figure 6A, ectopic expression of MAGE-C2 in HEK293T, SW480, HeLa and MCF7 cells stimulated cell proliferation. Meanwhile, suppression of MAGE-C2 expression in A375 cells decreased the cell proliferation (Figure 6B). In addition, we examined DNA synthesis in the S phase using FITC BrdU Flow kit in A375 cells with stable knockdown of MAGE-C2. As shown in Figure 6C, knockdown of MAGE-C2 reduced the ratio of Brdu-positive cells.

We further confirmed the above results by cell counting analysis. Stable MAGE-C2-overexpressing B16-GFP-MAGE-C2 cells grew faster over a 6 day period when compared to control cells (Figue 6D), while stable MAGE-C2 knockdown A375 cells grew more slowly than control cells (Figure 6E).

### Expression level of cyclin E correlates with MAGE-C2 expression in malignant melanoma tissues

**Figure 7:**
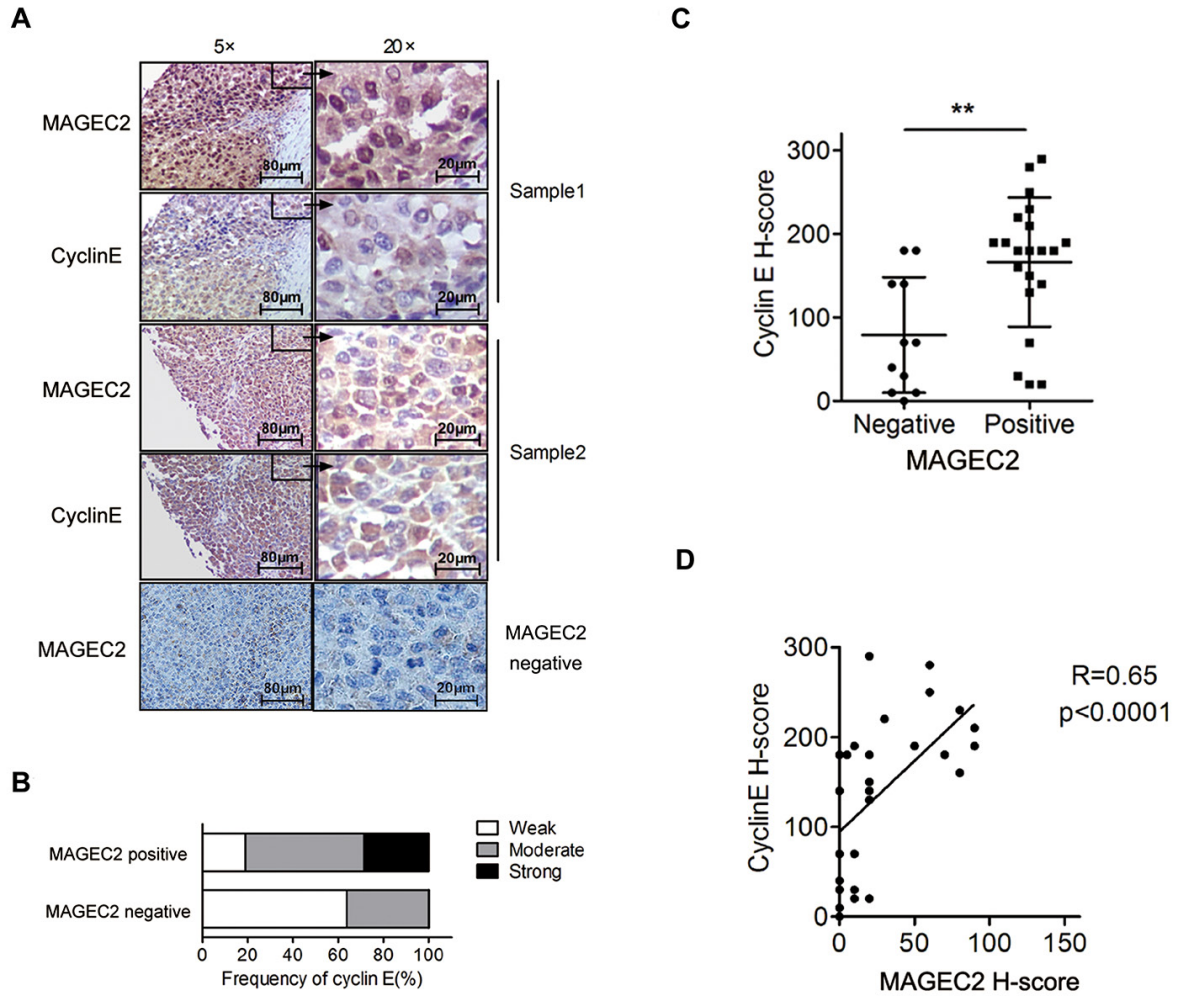
Expression levels of cyclin E correlate with MAGE-C2 expression in malignant melanoma tissues **A.** Examples of IHC for the MAGE-C2 or cyclin E in melanoma samples. **B.** and **C.** Distribution of cyclin E expression levels in the MAGE-C2-positive and MAGE-C2-negative tissues. **D.** Pearson correlation analysis of the expression levels of cyclin E and MAGE-C2.

We detected the expression of cyclin E and MAGE-C2 using IHC in human malignant melanoma tissues to analyze the correlation between the two molecules. Owing to much melanin distributing in 8 tissues, only 32 out of 40 samples were suitable for analysis. Cyclin E was detectable in 31 of 32 (96.88%) cases, while MAGE-C2 was observed in 21 of 32 tissues (65.63%). Examples of expression of MAGE-C2 or cyclin E are depicted in Figure 7A. The expression levels of cyclin E were found to be significantly higher in MAGE-C2-positive tumors than that in MAGE-C2-negative tumors (Figure 7B and 7C). Pearson correlation test showed that the expression of cyclin E correlates positively with MAGE-C2 expression (Figure 7D).

These data indicate that accumulation of cyclin E in malignant melanoma cells may result from decreased degradation.

## DISCUSSION

We identified cancer-testis antigen MAGE-C2 as a novel binding partner of Rbx1 and Cullin1, and a participant of Skp1-Cullin1-Rbx1-F box (SCF) complex. Furthermore, MAGE-C2 is involved in increasing cyclin E stability and cell cycle progression by inhibiting ubiquitin-mediated degradation of cyclin E. Our results suggest that MAGE-C2 may promote proliferation of cancer cells at least in part through protecting the protein stability of cyclin E.

Cyclin E is the best characterized Fbw7 substrate and a key component of the cell cycle machinery frequently deregulated in cancer [24–26]. Our findings indicate that MAGE-C2 not only binds with SCF complex, but also inhibit the ubiquitylation activity of SCF E3 ubiquitin ligase. Knockdown of MAGE-C2 results in increased turnover of cyclin E by promoting the ubiquitylation of cyclin E. Furthermore, relatively higher expression of cyclin E was also detected in MAGE-C2-positive melanoma tissues compared with MAGE-C2-negative samples.

Here, we evaluate the effects of knockdown or overexpression of MAGE-C2 on the cell cycle progression at G-S phase. We discover that MAGE-C2 can promote cell cycle progression, which is consistent with cyclin E functioning as a stimulator of the G1-S transition [27–28]. Furthermore, we also found that the expression of MAGE-C2 changes during cell cycle progression, with almost the same pattern as cyclin E (Supplementary Figure S5).

Since increased cyclin E abundance leads to genomic instability and tumorigenesis, its regulation has been extensively investigated [29]. Disrupted proteolysis is an important mechanism that deregulates cyclin E in cancers. Cyclin E accumulation in tumor cells is often caused by mutations in, or down-regulation of Fbw7 [26, 30–31]. Our data suggest another mechanism whereby MAGE-C2 can decrease SCF ubiquitin ligase activity and lead to the cyclin E accumulation in tumor cells.

The previous report by Doyle JM et al. indicated that MAGE-C2 enhances p53 degradation by increasing the E3 ubiquitin ligase activity of Trim28 [13]. However, our present study indicates MAGE-C2 decreases the activity of SCF E3 ligase. Taken together, we speculate that MAGE-C2 may exert distinct regulation mode for different E3 ligases. However, further studies will be necessary to understand the mechanism of MAGE-C2 in regulating different E3 ubiquitin ligases.

Since overexpression of cyclin E promotes cancer development, our work suggests that MAGE-C2 may play an oncogenic activity by decreasing cyclin E ubiquitylation and maintaining cyclin E at a level that favor cell cycle progression in cancer cells. A MAGE-C2 inhibitor may be useful in cancer therapies for MAGE-C2-expressing tumors.

## MATERIALS AND METHODS

### Plasmids and siRNA

pEGFP-C1-MAGE-C2 plasmid was provided by Boquan Jin (Forth Military Medical University, Xi'an, China). The expression vectors pRK-FLAG-MAGE-C2, pRK-HA-MAGE-C2, and pCMV-(HA-Ub)_4_ have been described previously [32]. To generate pGEX-4T-2-MAGE-C2, pRK-FLAG-MAGE-C2 was digested with SalI/NotI and the resulting fragment was cloned into pGEX-4T-2. pCMV-FLAG-Cullin1 was provided by Xiaoyan Qiu (Peking University Health Science Center). pCMV-FLAG-Cullin1 was used as templates in PCR to generate NotI/BamHI Cullin 1 full length or truncated cDNA fragments to construct pRK-FLAG-Cullin1, pcDNA3.1-myc-Cullin1 (wt), -(ΔN), -(ΔC), and -(ΔM). The cDNAs encoding human Rbx1 and Skp1 were generated by RT-PCR and subcloned into SalI/NotI sites on the pRK-FLAG, pRK-HA and pGEX-4T-2 vectors. The expression vectors of pcDNA3-myc-Skp2 and pcDNA3-myc-Fbw7 were obtained from Luyang Sun (Peking University Health Science Center) and Xin Ye (Institute of microbiology Chinese Academy of Science), respectively. To construct pRK-FLAG-Skp2 and pRK-FLAG-Fbw7, pcDNA3-myc-Skp2 or pcDNA3-myc-Fbw7 was used as a template in a PCR to generate a SalI/NotI Skp2 or Fbw7 cDNA fragment. Expression vectors for MAGE-C2 deletion mutants (MAGE-C2 Δ31–147, MAGE-C2 Δ148–314, MAGE-C2 Δ245–373) were constructed in pRK-HA by PCR from pRK-HA-MAGE-C2. The siRNA sequences for MAGE-C2 and control were described previously [32]. Cullin1 siRNA: 5′-GGUUAUAUCAGUUGUCUAA-3′.

### Cell culture and treatments

All cells were maintained in Dulbecco's modified Eagle medium (DMEM) supplemented with 10% fetal bovine serum and antibiotics. The stable cell lines B16-GFP-MAGE-C2 (overexpressing MAGE-C2) and B16-GFP (overexpressing GFP) were established previously [21].

Stable knockdown of MAGE-C2: The pGPU6/Neo-shRNA vector was used to express MAGE-C2 specific and control shRNAs. Target sequences are as follows: MAGE-C2 shRNA (5′-CAATTGATACCGCAGATGA-3′), Control shRNA (5′-TTCTCCGAACGTGTCACGT-3′). A375 cells were transfected with pGPU6/Neo-shMAGE-C2 or pGPU6/Neo-shControl vectors and stable populations were selected using 1 mg/ml G418. The knockdown effect was evaluated by Western blotting.

For G1 phase cell cycle synchronization, A375 and B16 cells were treated with 20 μM lovastatin (Sigma, St Louis, MO, USA) for 34 h, and then the medium was removed and replaced with fresh medium containing 2 mM mevalonate. At the indicated time intervals following mevalonate addition cells were harvested, and stained with propidium iodide (PI) staining solution (0.24 mg/ml PI, 1% Triton-X 100, 5 mg/ml RNase in PBS) for 15 min. DNA content was measured using flow cytometric analysis.

To determine cyclin E protein stability, A375 cells were transfected with siRNA. At 24 h post-transfection, cells were treated with vehicle (DMSO) or MG132 (20 μM) (Sigma) for 8 h before addition of CHX (50 μg/ml), and then incubated at 37°C for different time points and subsequently harvested for immunoblotting.

### Antibodies

Antibodies against CDK2, CDK6, cyclin D1, cyclin B, myc, HA, and Flag were purchased from MBL (Nagoya, Japan). Rabbit anti-Cullin1, anti-Skp1, and anti-cyclin E antibodies were from Santa Cruz (Dallas, TX, USA). Mouse anti-Skp1 and anti-Cullin1 antibodies were from Invitrogen (Carlsbad, CA, USA). Anti-Fbw7 antibody was from Sigma. Anti-Rbx1 antibody was from Cell Signaling Technology (Beverly, MA, USA). Anti-MAGE-C2 antibodies have been described previously (25). Antibodies specific for GAPDH, GFP and GST were from ProteinTech (Chicago, Illinois, USA). Mouse or rabbit IgG (Sigma) was used as control.

### GST pull-down assays and *in vitro* translation

Recombinant human MAGE-C2 protein was prepared as previously described [21]. Skp1 protein was purchased from abcam (Cambridge, UK). GST, GST-MAGE-C2, and GST-Skp1 were expressed in bacterial host BL21 (DE3) and purified with glutathione Sepharose 4B beads (GE Healthcare Life Sciences, Pittsburgh, PA, USA).

*In vitro* translates of pcDNA3.1-myc-Cullin1 (wt), -(ΔC), -(ΔN), and -(ΔM) were produced using the TNT Quick rabbit reticulocyte lysate system (Promega, Madison, WI, USA). For GST pull-down assay, approximately 10 μg glutathione Sepharose-linked GST or GST-fusion protein was incubated with MAGE-C2, Skp1, or Cullin1 proteins in 200 μl of GST binding buffer (PBS with 0.1% NP-40, 5 mM dithiothreitol and protease inhibitors cocktail) with rotation overnight at 4°C. The beads were then washed, and bound proteins were separated by SDS-PAGE and detected with Western blotting.

### Immunoprecipitation (IP) and immunoblotting

IP and immunoblotting experiments were performed as previously described [32]. Briefly, the cells were lysed in IP buffer, and the cell lysates were incubated with appropriate antibodies and protein A-sepharose beads at 4°C for 2–3 h. The IP beads were then washed, and bound proteins were analyzed by immunoblotting. GAPDH was used as a control.

### *In situ* proximity ligation assay (*in situ* PLA)

A375 cells were grown directly on glass coverslips for 24 h, and then fixed with 3.7% formaldehyde in PBS, and further permeabilized by methanol. After blocking in PBS-5% skimmed milk, cells were incubated with combinations of the primary mouse anti-MAGE-C2/rabbit anti-Cullin1, mouse anti-MAGE-C2/rabbit anti-Rbx1, or mouse anti-MAGE-C2/rabbit Skp1 overnight at 4°C. *In situ* protein bindings were detected using Duolink *in situ* PLA kit according to the manufacturer's instructions (Sigma). Cells were stained with Hoechst to visualize nuclei. The resulting spots were visualized using confocal microscopy.

### RNA extraction and real-RT-qPCR

Total RNA was extracted from cultured cells with Trizol reagent (Life Technologies, Carlsbad, CA, USA) according to the manufacturer's instructions. cDNA was synthesized using Reverse Transcription Kit (Promega). Expression of mRNA was quantified using real-time PCR performed in duplicate with SYBR Green qPCR Master Mix (Promega). The abundance of transcripts of interest was normalized against that of β-actin as an internal standard.

### Ubiquitylation assays

The *in vivo* ubiquitylation assay has been described previously [32]. Briefly, GFP or GFP-MAGE-C2 and HA-Ub were co-expressed in HEK293T cells. At 36 hours post-transfection, cells were treated with MG132 (20 μM) for 8 hours and then cell extracts were subjected to immunoprecipitation with anti-cyclin E or anti-HA antibodies, and copurified proteins were immunoblotted with appropriate antibodies.

### Cell proliferation

[^3^H] thymidine incorporation assay: After 24 hours transfection, the cells were seeded in 96-well culture plates in triplicate and cultured for 48 hours. [^3^H] thymidine (Beijing Atomic Energy Institute, China) was added at 0.5 μCi/well 8 hours before the end of culture. Cells were collected and the counts/min value was determined with scintillation fluid on a β-counter.

Cell growth rate determination: cells were seeded in 6-well culture plates at a low density. At the indicated times, cells in triplicate wells were trypsinized and counted.

For BrdU labeling, cells were incubated with 10 μM 5-bromo-2′-deoxy-uridine (BrdU) for 30 min before staining with anti-BrdU-FITC antibody and 7-AAD (FITC BrdU Flow kit, BD Pharmingen, San Diego, CA, USA), then analyzed with a FACS Calibur flow cytometer and CellQuest software.

### Immunohistochemistry (IHC)

The expressions of MAGE-C2 and cyclin E were analyzed immunohistochemically using melanoma tissue array (ME481a, US Biomax, Rockville, MD, USA). Two consecutive 5 μm-thick paraffin-embedded tissue sections were deparaffinized and rehydrated. Endogenous peroxidase was blocked with 3% H_2_O_2_. Antigen retrieval was performed in solution of 1 mM EDTA and 10 mM Tris (pH: 9.0) at 95°C for 60 min. Normal goat serum (5%) was applied to block any nonspecific protein binding sites. Sections were incubated with anti-MAGE-C2 or anti-cyclin E antibodies at 4°C overnight, followed by adding dextran carrying anti-rabbit IgG conjugated to horseradish peroxidase (HRP). Positive staining was developed using Dako REAL EnVision detection system (Dako, Carpinteria, CA, USA).

Immunohistochemistry evaluations were performed by one pathologist and one trained reader who were blinded to the experimental data. The immunohistochemical staining of cyclin E and MAGE-C2 was scored (H score) by taking into account both the intensity of staining and the percentage of positive cells [33]. Based on the value of H-score, expression level of cyclin E was classified into 3 categories: weak (H-score ≤ 100), moderate (100 < E H-score < 200) and strong (H-score ≥ 200).

### Statistical analysis

All the experiments were repeated at least twice or three times. Quantitative results are presented as mean ± SD (standard deviation). Comparisons between two independent groups were made by Student's *t* test. Differences were considered statistically significant at *P* < 0.05.

## SUPPLEMENTARY FIGURES


