# Supplementary material for: Cancer-testis antigen MAGE-C2 binds Rbx1 and inhibits ubiquitin ligase-mediated turnover of cyclin E
Source: Oncotarget. 2015 Oct 19;6(39):42028–39. doi: 10.18632/oncotarget.5973 (PMC4747207; doi:10.18632/oncotarget.5973)
Supplement: Supplementary file 1 [file oncotarget-06-42028-s001.pdf]

SUPPLEMENTARY FIGURES

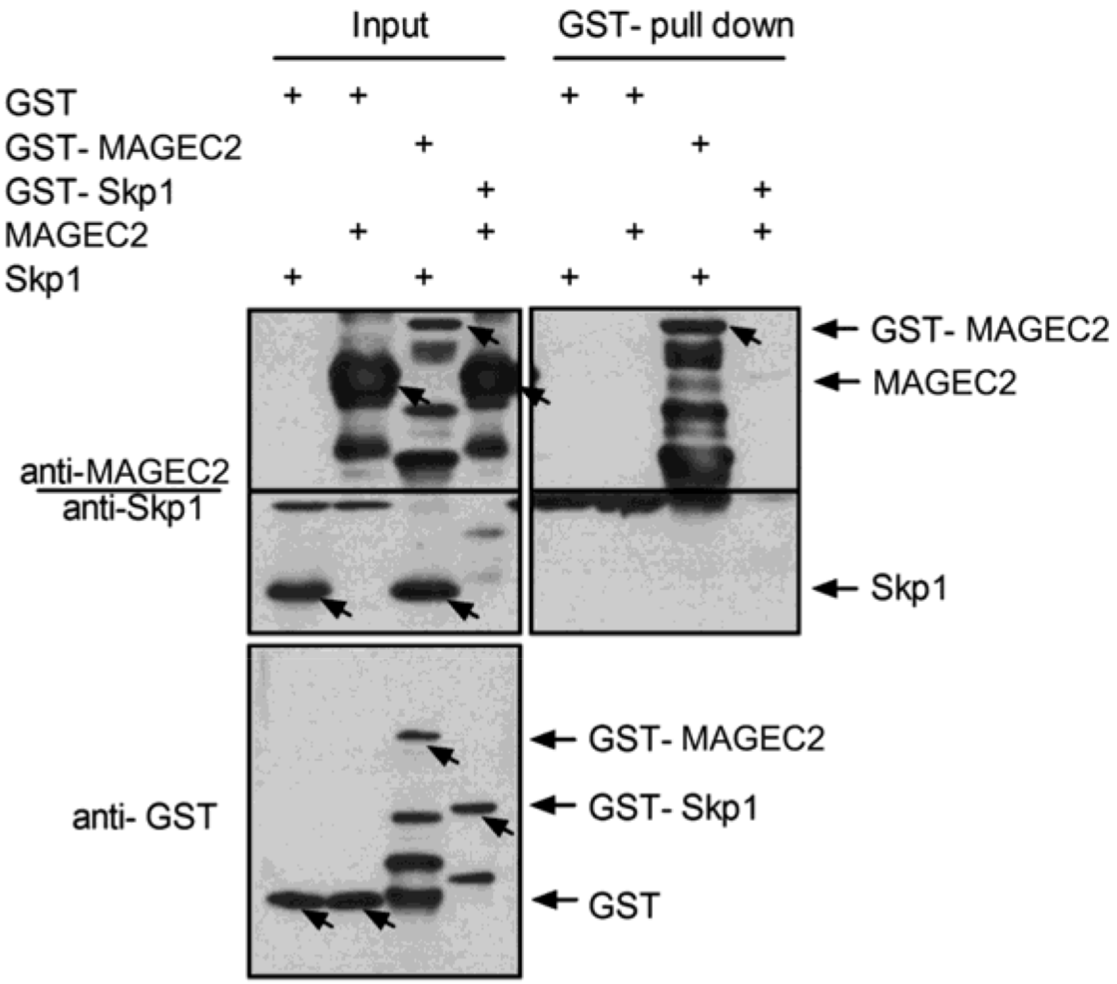

Supplementary Figure S1: MAGE-C2 does not interact directly with Skp1. GST pull-down experiment was performed with GST-MAGE-C2 or GST-Skp1 and purified Skp1 or MAGE-C2. Proteins were detected with Western blotting.

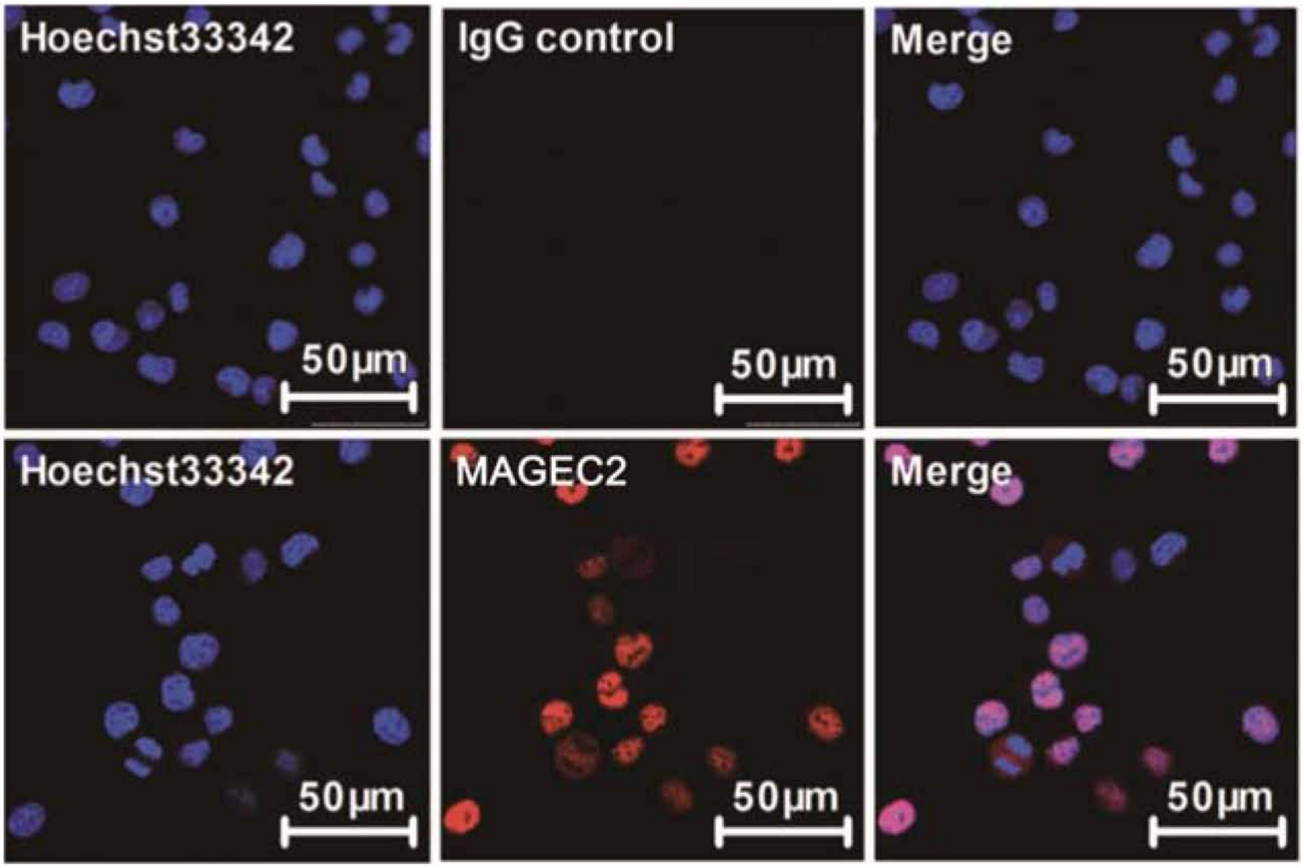

Supplementary Figure S2: MAGE-C2 is localized in the nucleus. Distribution of MAGE-C2 in A375 cells was detected with anti-MAGE-C2 monoclonal antibody by indirect immunofluorescence staining. The cells were stained with Hoechst to visualize the nuclei. IgG was used as a negative control.

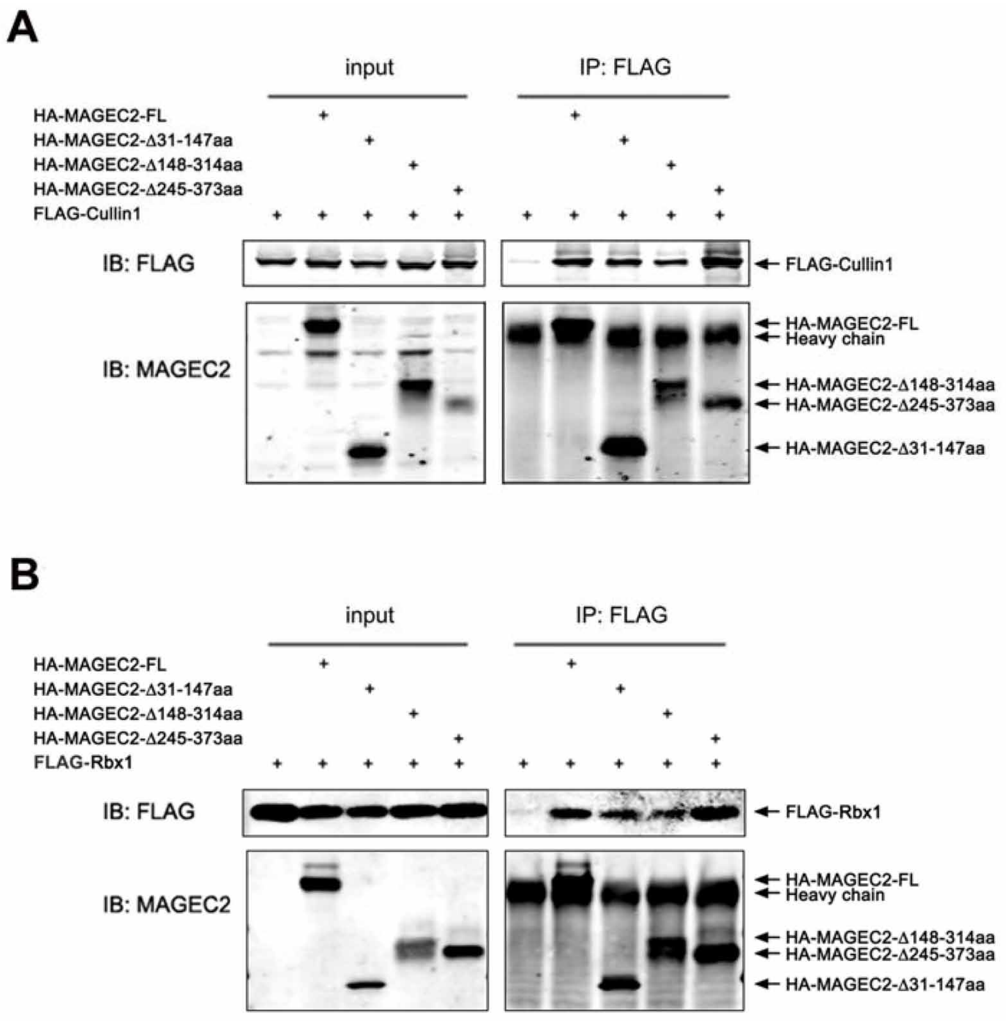

Supplementary Figure S3: Multiple Cullin1/Rbx1 binding sites on MAGE-C2. A. Binding regions of MAGE-C2 to Cullin1. **B.** Binding regions of MAGE-C2 to Rbx1. Full length (FL) or deletion mutants of HA-tagged MAGE-C2 were cotransfected with FLAG-Cullin1 or FLAG-Rbx1 into HEK293T cells. Cell lysates were sub jected to immunoprecipitation with anti-FLAG antibody followed by immunoblotting with anti-FLAG or anti-MAGE-C2 polyclonal antibodies.

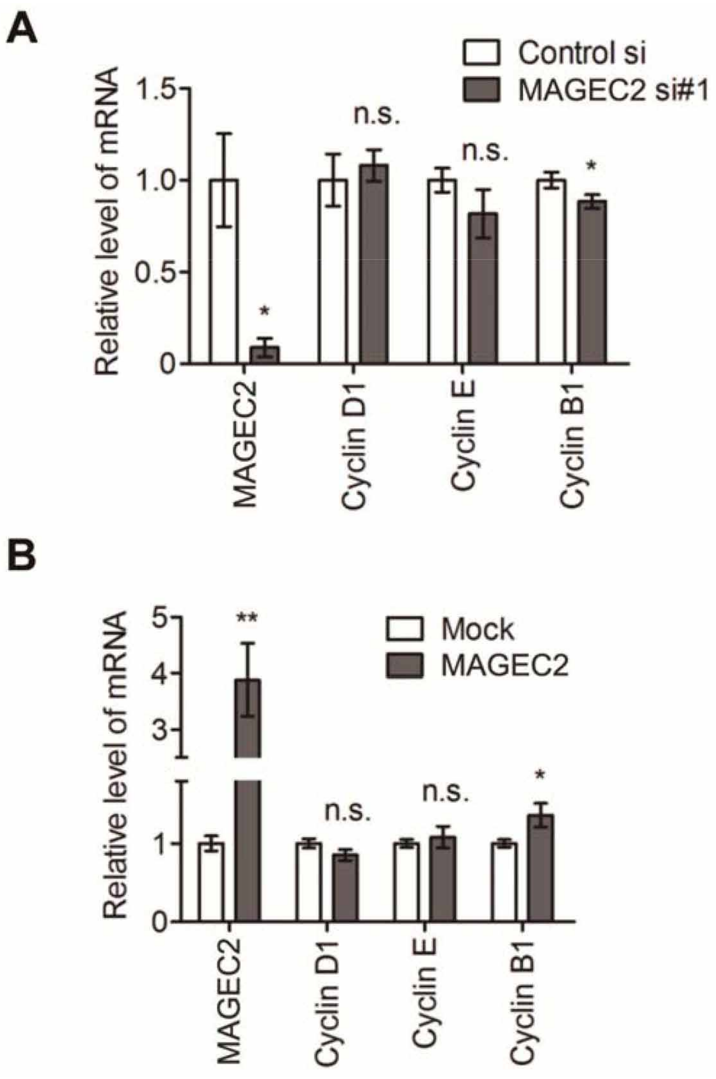

Supplementary Figure S4: Effects of MAGE-C2 expression on cyclin E mRNA expression. A. Relative mRNA levels of cyclin E, cyclin D1, cyclin B1, and GAPDH in A375 cells with (MAGE-C2 siRNA) or without (control). MAGE-C2 depletion was measured by quantitative reverse transcription-PCR analysis. The abundance of the mRNA of interest was normalized against that of GAPDH as an internal standard. Columns, mean of triplicate analyses of three different samples; bars, SD. **B.** Relative mRNA levels of cyclin E, cyclin D1, cyclin B1, and GAPDH in HEK293T cells transfected with MAGE-C2-expressing or control (mock) vectors.

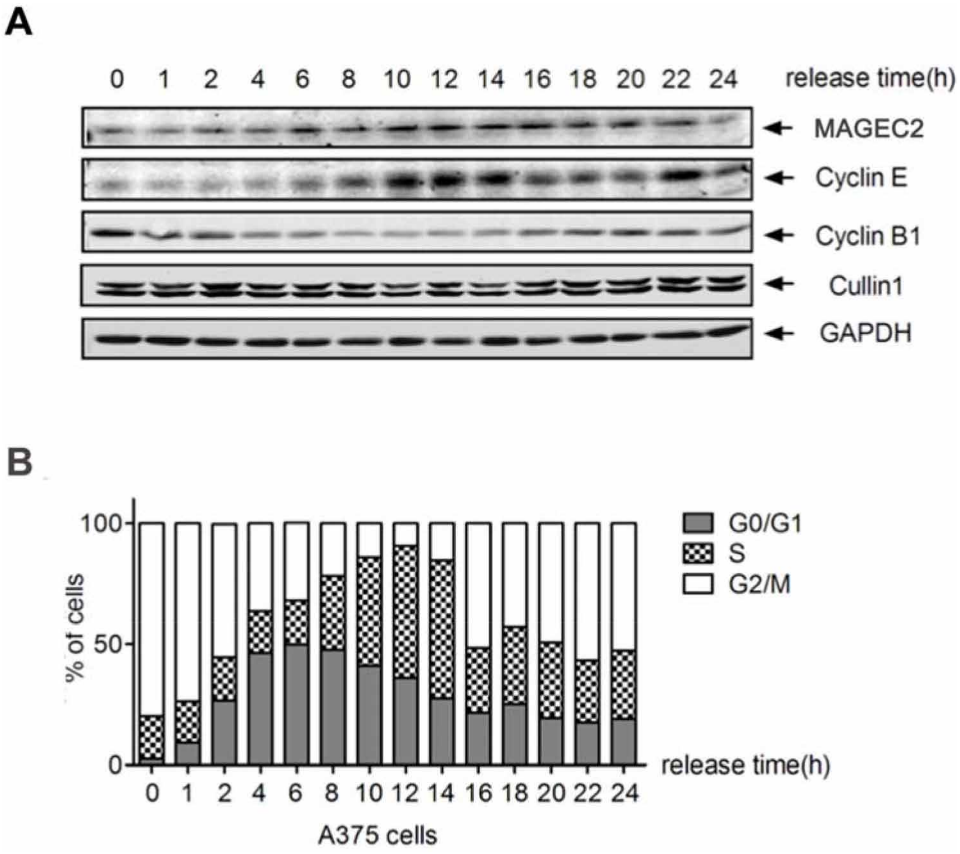

Supplementary Figure S5: Expression of MAGE-C2 changes during cell cycle progression. A. Expression of MAGE-C2 protein in A375 cells following release of a nocodazole-induced M-phase block. Cells were harvested at indicated time intervals, and the cell lysates were immunoblotted by indicated antibodies. GAPDH was used as a control. **B.** The number of cells (in percentage) in G0/G1, S, and G2/M at different times after block release. Cells were harvested at indicated time points and cellular DNA contents were analyzed by FACS.
